# Effect of 3% saline and furosemide on biomarkers of kidney injury and renal tubular function and GFR in healthy subjects – a randomized controlled trial

**DOI:** 10.1186/s12882-019-1342-x

**Published:** 2019-06-03

**Authors:** F. H. Mose, A. N. Jörgensen, M. H. Vrist, N. P. Ekelöf, E. B. Pedersen, J. N. Bech

**Affiliations:** 1 Holstebro Hospital, Hospital Unit West, Holstebro, Denmark; 2 University Clinic in Nephrology and Hypertension, Aarhus University, Aarhus, Denmark; 3 Department of Anaesthesiology, Holstebro Hospital, Hospital Unit West, Holstebro, Denmark

**Keywords:** Hypertonic saline, 3% saline, Hyperchloremic acidosis, NGAL, KIM-1, Fractional excretion of sodium

## Abstract

**Background:**

Chloride is speculated to have nephrotoxic properties. In healthy subjects we tested the hypothesis that acute chloride loading with 3% saline would induce kidney injury, which could be prevented with the loop-diuretic furosemide.

**Methods:**

The study was designed as a randomized, placebo-controlled, crossover study. Subjects were given 3% saline accompanied by either placebo or furosemide. Before, during and after infusion of 3% saline we measured glomerular filtration rate (GFR), fractional excretion of sodium (FE_Na_), urinary chloride excretion (u-Cl), urinary excretions of aquaporin-2 (u-AQP2) and epithelial sodium channels (u-ENaC_γ_), neutrophil gelatinase-associated lipocalin (u-NGAL) and kidney injury molecule-1 (u-KIM-1) as marker of kidney injury and vasoactive hormones: renin (PRC), angiotensin II (p-AngII), aldosterone (p-Aldo) and arginine vasopressin (p-AVP). Four days prior to each of the two examinations subjects were given a standardized fluid and diet intake.

**Results:**

After 3% saline infusion u-NGAL and KIM-1 excretion increased slightly (u-NGAL: 17 ± 24 during placebo vs. -7 ± 23 ng/min during furosemide, *p* = 0.039, u-KIM-1: 0.21 ± 0.23 vs − 0.06 ± 0.14 ng/ml, *p* <  0.001). The increase in u-NGAL was absent when furosemide was given simultaneously, and the responses in u-NGAL were not significantly different from placebo control. Furosemide changed responses in u-KIM-1 where a delayed increase was observed. GFR was increased by 3% saline but decreased when furosemide accompanied the infusion. U-Na, FE_Na_, u-Cl, and u-osmolality increased in response to saline, and the increase was markedly pronounced when furosemide was added. FE_K_ decreased slightly during 3% saline infusion, but simultaneously furosemide increased FE_K_. U-AQP2 increased after 3% saline and placebo, and the response was further increased by furosemide. U-ENaC_γ_ decreased to the same extent after 3% saline infusion in the two groups. 3% saline significantly reduced PRC, p-AngII and p-Aldo, and responses were attenuated by furosemide. p-AVP was increased by 3% saline, with a larger increase during furosemide.

**Conclusion:**

This study shows minor increases in markers of kidney injury after 3% saline infusion Furosemide abolished the increase in NGAL and postponed the increase in u-KIM-1. The clinical importance of these findings needs further investigation.

**Trial registration:**

(EU Clinical trials register number: 2015–002585-23, registered on 5th November 2015)

## Background

In critically ill patients and patients undergoing surgery intravenous fluid treatment is an important part of maintaining cardiovascular homeostasis. Crystalloids and colloids are widely used as fluid resuscitation [1–3]. Crystalloids differ in electrolyte composition. Crystalloids with a high content of sodium and chloride such as isotonic saline induce hyperchloremic metabolic acidosis compared to solutions with a lower sodium and chloride content, particularly when administered in higher doses [4–7]. Chloride and hyperchloremic acidosis may impair renal blood flow and induce kidney injury [4, 8–11]. This was first demonstrated in animal experiments, where high chloride concentration during renal perfusion was associated with increased renal vasoconstriction and reductions in renal blood flow and glomerular filtration rate [9, 11]. In healthy subjects isotonic saline compared to infusion with fluids with lower sodium and chloride contents decreased renal blood flow (RBF). [10] In patients submitted to an emergency department, infusion of low chloride containing solutions was associated with a lesser degree of AKI compared to fluid solutions with a higher chloride content [4, 7]. In the clinical setting however the importance of dyschloremia and infusion of high chloride containing solutions is still under much debate [12–14].

In daily practice plasma creatinine is used to estimate renal function. In case of acute kidney injury (AKI) changes in creatinine are seen within days. Novel biomarkers such as neutrophil gelatinase-associated (NGAL) and kidney injury molecule-1 (KIM-1) are within hours able to detect kidney injury and predict the risk of renal replacement therapy and chronic kidney disease (CKD). [15–19] KIM-1 is produced in the proximal tubulus and NGAL in the distal tubulus, and can both be detected in the urine during very little kidney injury [19].

We therefore hypothesized that a large load of chloride given as 3% saline will induce hyperchloremic acidosis and a subsequent kidney injury, which can be detected by measuring glomerular filtration rate (GFR), renal tubular function, and biomarkers of AKI in the urine. In addition, we hypothesized that furosemide impairs kidney damage induced by 3% saline.

We investigated these hypotheses in a study designed as a randomized, placebo-controlled, crossover study were subjects were given 3% saline accompanied by either placebo or furosemide on two separate occasions, where renal function, urinary excretion of biomarkers of kidney injury, and plasma concentrations of vasoactive hormones were measured.

## Methods

### Subjects

Screening examination included physical examination, medical history, ECG, office BP, clinical biochemistry and urinary albumin analysis.

### Inclusion criteria

Healthy women and men, age 18–40 years, BMI 18.5–30.0 kg/m^2^. Exclusion criteria: History with or clinical signs of diseases in the central nervous system, lungs, thyroid gland, heart, liver or kidneys, diabetes mellitus or malignancies. Clinical important deviations in screening blood or urinary samples, office blood pressure > 140 mmHg systolic and/or > 90 mmHg diastolic, nursing or pregnancy, alcohol or drug abuse, smoking, allergy or intolerance towards furosemide or unwillingness to participate,. Withdrawal criteria: Symptoms of hypotension or office BP repeatedly below 50 mmHg diastolic and/or 90 mmHg systolic. Development of exclusion criteria.

### Design

The study was a placebo-controlled, randomized, single-blinded, crossover trial. After inclusion subjects were allocated to treatment via computer-generated randomization in blocks of six. Consequently, the subjects received glucose (placebo) or furosemide in a random order on 2 separate examination days. Awashout period of at least 14 days was required between examinations.

### Study drugs

Hypertonic saline (3% NaCl, Skanderborg Pharmacy, Skanderborg, Denmark) was given intravenously as continuous infusion (7 ml/kg/hour) for 60 min. Furosemide (Furix, 2 ml of 10 mg/ml, Takeda Pharma, Osaka, Japan) and isotonic glucose (2 ml 50 g glucosemonohydrate/l, Fresenius, Bad Homburg vor der Höhe, Germany) were identical in appearance to the study subjects. Furosemide was administered at a dose of 20 mg (2 ml).

### Effect variables

The primary effect variable was u-NGAL. Secondary effect variables were free water clearance (C_H2O_)_,_ GFR, (fractional excretion of sodium) FE_Na_, (fractional excretion of potassium) FE_K,_ u-albumin, u-KIM-1, urinary excretions of aquaporin-2 (u-AQP2) and epithelial sodium channels (u-ENaC_γ_), plasma and urinary osmolality, plasma concentration of renin (PRC), angiotensin II (p-AngII), aldosterone (p-Aldo) and vasopressin (p-AVP), brachial systolic and diastolic blood pressure (DBP, SBP) and heart rate (HR).

### Recruitment

Subjects were consecutively recruited by announcements in local newspapers in community Holstebro, Denmark. After written and oral information that included safety concerns by 3% saline and furosemide infusion, a written consent was obtained. A clinical history was gives and examination was performed, blood and urine samples were drawn and ECG was performed to ensure that the subject fulfilled the inclusion criteria and did not meet exclusion criteria.

### Number of subjects

With a significance level of 5% and a power of 80% a total of 23 subjects were needed to detect an 85 ng difference in u-NGAL (SD 144 ng). During examination incomplete voiding was expected in some participants. Hence we estimated that 27 subjects should complete the study.

### Experimental procedure

Examinations were carried out after 4 days of standardized diet and fluid intake [20–23]. The diet comprised three main meals and three minor meals. Subjects were instructed to eat variedly from the diet until satiated. The diet contained 11,000 kJ/day, was composed of 55% carbohydrates, 15% protein and 30% fat, and ensured a sodium intake of 150 mmol daily. Fluid intake was 2.5 L per day. Two cups of tea or coffee were allowed daily. No alcohol consumption was allowed.

Collection of 24-h urine samples were performed before each examination. The 24-h urine collection was analyzed for sodium, potassium, chloride, osmolality, creatinine, albumin, AQP2, ENaC_γ_, NGAL and KIM-1.

After an overnight fast, subjects arrived at 8 AM. Two indwelling catheters for blood sampling and administration of 3% saline and furosemide or glucose (placebo) and ^51^Cr-EDTA, were placed in cubital veins, one in each arm. Every 30 min after arrival, participants received an oral water load of 175 ml. Subjects were kept in a supine position in a quiet, temperature-controlled room (22–25 °C). Only exception from the supine position was that when urine was collected by voiding in sitting or standing position. At 10.30 AM 3% saline was given as a continuous infusion for 60 min (7 ml/kg/hour) [20]. Furosemide (20 mg in 2 ml) or glucose (2 ml) was given at 10.30 AM according to randomization.

Blood and urine samples were collected every 30 min from 9:30 AM to 2.30 PM, except for the period between 11 and 12 AM and 1.30 PM to 2.30 PM, blood and urine only was collected once. Urine collections were analyzed for potassium, sodium, chloride, ^51^Cr-EDTA, creatinine, osmolality, AQP2, ENaC_γ_, NGAL and KIM-1. The first three clearance periods from 9:00 AM to 10.30 AM were defined as baseline period.

Blood samples were drawn at 10.30 AM (baseline), 11.30 AM (after 60 min of 3% saline infusion), and at 1 PM (90 min after termination of infusion) for determination of p-AVP, p-Aldo, PRC and p-Ang II.

Urinary spot samples were collected 1 and 3–5 days after the examination days. These samples were analyzed for potassium, sodium, chloride, creatinine, osmolality, NGAL, KIM-1, AQP2 andENaC_γ_,.

### Blood pressure measurements

Office BP used at inclusion was measured using the semiautomatic, oscillometric device, Omron 705IT (Omron Matsusaka CO. Ltd., Matsusaka City, Japan). BP during examination were measured using the automatic oscillometric device, Mobil-O-Graph PWA (Medidyne A/S, Nærum, Denmark). BP was measured as double measurements every 30 min from 9:30 AM to 2.30 PM, except for the period between 11 and 12 AM and 1.30 PM to 2.30 PM, where blood pressure only was measured once. The first 4 measurements were defined as baseline.

### Biochemical analyses

Urine samples were stored frozen at − 20 °C until analyzed. U-AQP2 and u-ENaC_γ_ were measured by using radioimmunoassays (RIA) as previously described [20–25]. Antibodies were raised in rabbits to synthetic peptides for AQP2 and ENaC_γ_ as previously described [20, 23, 26, 27]. The antibodies against AQP2 and ENaC_γ_ was a gift from Professor Robert Fenton and Professor Søren Nielsen and, The Water and Salt Research Center, Institute of Anatomy, Aarhus University, Denmark.

Blood samples collected for measurements of vasoactive hormones were centrifuged and plasma was separated, and kept frozen until assayed as previously described [26]. AVP and Ang II were extracted from plasma and then determined by RIA [26, 28, 29]. PRC was determined by immunoradiometric assay as previously described [26]. Aldo was determined by RIA as previously described [26].

A commercial enzyme-linked immunosorbent assay (ELISA) from Bioporto (Hellerup, Denmark) was used to determine the u-NGAL [30]. Minimal detection level was 1.4 pg/ml. Variations were interassay max 8% and intraassay max 14%.U -KIM-1 was determined with a commercial enzyme-linked ELISA-kit (Quantijine ELISA) from R&D Systems. Minimal detection level was 3.0 pg/ml. Variations were interassay max 7.8% and intraassay max 4.4% All samples were analyzed with kits from the same batch.

GFR was estimated using constant infusion clearance technique with ^51^Cr-EDTA as reference marker. A GFR variation og 15% variation or more between the three baseline periods led to the exclusion of clearance related analysis [20, 22].

Urine and plasma concentration of potassium, sodium, chloride, creatinine, albumin and were determined at the Department of Clinical Biochemistry by routine methods.

### Calculations

C_H2O_ was calculated with the formula C_H2O_ = UO – C_osm_, where C_osm_ is osmolar clearance and UO is urinary output.

FE_Na_ and FE_K_ were calculated using to the formula FE_X_ = (X_u_ * V / X_p_)/GFR. V is urine flow in ml/min and X_u_ and X_p_ are urine and plasma concentrations of X. In 24-h urine creatinine clearance was used as an estimation of GFR.

### Statistics

Data are presented as means ± standard deviations (SD), when normality was present. If normality was not presenta data are presented as medians with 25 and 75% percentiles in brackets. A paired comparison between and within groups was performed with paired t-test or Wilcoxon signed rank test. To test for deviation during experimental procedure a general linear model for repeated measures (GLM) was performed. If data did not show normality they were logarithmic transformed before GLM. Friedman’s test was used to test for deviations within treatment of vasoactive hormones. Correlations were performed with Pearson correlation. Statistical significance was defined as *p* <  0.05. Statistical analyses were performed using PASW version 20.0.0 (SPSS Inc.; Chicago, IL, USA).

## Results

### Demographics

Thirty-two subjects were screened for participation in the study. Exclusion was made for eight subjects due to anaemia (1) and withdrawal of consent (7). Thus, 24 patients were included and completed the trial. The 24 subjects (12 females, 12 males), had a mean BMI 23.7 ± 2.8 kg/m^2^, age 23 ± 5 years, office BP 123/70 ± 9/8 mmHg, p-creatinine 72 ± 13 μmol/L, urine albumin 8 (1;10) mg/L, p-hemoglobin 8.8 ± 0.8 mmol/L.

### GFR and tubular function during baseline conditions

In 24-h urinary collection made prior to the two examinations sodium (u-Na,) FE_Na_ and chloride (u-Cl) excretion rate was slightly but significantly lower prior to furosemide compared to placebo (Table 1). Urine output, C_H2O_, urinary excretions of potassium and creatinine, FE_K_, creatinine clearance, UAER, U-AQP-2, u-ENaC_γ,_ u-NGAL and u-KIM-1 were not significantly different between treatments (Table 1).

**Table 1:** 24-h urine collection prior to two examinations in a randomized, cross-over study of 24 healthy subjects

	Placebo	Furosemide	*P*-value
Urine output (mL/minute)	1.84 ± 0.36	1.73 ± 0.39	0.242
C_H2O_ (mL/minute)	−0.23 ± 0,61	−0.15 ± 0.38	0.436
U-creatinine (mmol/24 h)	15.5 ± 4.1	15.2 ± 4.0	0.917
Creatinine clearance (mmol/mL pr. m^2^)	134 ± 24	130 ± 19	0.753
U-Na (mmol/24 h)	124 ± 37	100 ± 28	0.017
FE_Na_ (%)	0.62 ± 0.19	0.57 ± 0.9	0.016
U-Cl (mmol/24 h)	128 ± 31	108 ± 27	0.052
U-K (mmol/24 h)	62 ± 14	62 ± 23	0.601
FE_K_ (%)	10.8 ± 2.3	11.1 ± 4.4	0.438
UAER (mg/24 h)	7 (4;10)	7 (5;9)	0.440
U-AQP-2/min (ng/minute)	0.81 ± 0.31	0.77 ± 0.20	0.562
U-AQP-2/creatinine (ng/mmol)	75 ± 15	76 ± 22	0.826
U-ENaC_γ_ / min (ng/minute)	0.79 ± 0.30	0.71 ± 0.25	0.430
U-ENaC_γ_ /creatinine (ng/mmol)	77 ± 30	70 ± 24	0.327
U-NGAL / min (ng/min)	16 (7;43)	15 (7;27)	0.063
U-NGAL /creatinine (ng/mmol)	1401 (649;4777)	1409 (524;3433)	0.109
U-KIM-1 / min (ng/min)	0.41 ± 0.21	0.41 ± 0.20	0.580
U-KIM-1 /creatinine (ng/mmol)	39 ± 23	40 ± 19	0.831

Urine output, *C*_*H2O*_ free water clearance, *U-Na* urine excretion of sodium, and *U-K* potassium, *FE*_*Na*_ fractional excretion of sodium, and *FE*_*K*_ potassium, creatinine clearance, *UAER* urinary excretions rates of albumin, *u-AQP-2/min* aquaporin-2, *u-ENaC*_*γ*_*/min* γ-fraction of the epithelial sodium channel, *u-NGAL/min* neutrophil gelatinase-associated lipocalin and *u-KIM-1/min* kidney injury molecule-1 and in relation to creatinine (u-AQP-2/creatinine, u-ENaC_γ_/creatinine, u-NGAL/creatinine, u-KIM-1/creatinine. Urine were collected from 07.00 am on the day before the day of examination day to 07.00 am on the day of examination. Data are shown as means ± SD in brackets or medians with 25 and 75 percentiles in brackets. Statistics are performed with paired t-test or Wilcoxon signed rank test

Similar results were found at baseline during examinations. At baseline during examinations urine output, C_H2O_, urinary excretions of potassium, FE_K_, GFR, UAER, U-AQP-2, u-ENaC_γ,_ u-NGAL and u-KIM-1 were similar between treatment arms (Tables 3, 4 and 5).

### Bodyweight

At baseline body-weight was similar on the two examinations (74.4 ± 11.9 kg before placebo vs 73.8 ± 11.3 kg before furosemide, *p* = 0.860). After 3% saline and placebo body-weight increased to 74.9 ± 11.9 kg (*p* <  0.001) and when furosemide was given simultaneously body-weight decreased to 72.8 ± 11.3 kg (*p* <  0.001). The two responses in bodyweight were significantly different between treatments (0.5 ± 0.4 kg vs. -1.0 ± 0.5 kg, *p* <  0.001).

### Plasma electrolytes

Plasma-Na, p-Cl, p-K, p-osmolality and p-total carbon dioxide were similar at baseline. (Table 2). Plasma-Na, p-Cl and p-osmolality increased after 3% saline. Furosemide did not change the response to 3% saline regarding p-Na, but the increase after 3% saline was less pronounced for p-Cl and increased for p-osmolality when furosemide was given (*p* <  0.001).

**Table 2:** Effect of hypertonic saline and furosemide on plasma concentrations of electrolytes in a randomized, cross-over study of 24 healthy subjects

Period	Baseline (90 min)	After 60 min hypertonic saline infusion (150 min)	90 min post hypertonic saline infusion (240 min)	*P*-value (difference in response)
p-Na (mmol/L)				
Placebo	140 ± 2	144 ± 2^*^	141 ± 2^*^	0.073
Furosemide	139 ± 2	144 ± 2^*^	141 ± 2^*^	
p-K (mmol/L)				
Placebo	3.8 ± 0.2	3.7 ± 0.2^*^	4.0 ± 0.2^*^	0.001
Furosemide	3.7 ± 0.2	3.5 ± 0.2^*, †^	3.8 ± 0.2^†^	
p-Cl(mmol/L)				
Placebo	105 ± 2	111 ± 2^*^	107 ± 2^*^	< 0.001
Furosemide	104 ± 2	108 ± 2^*, †^	104 ± 2^*, †^	
p-Osmolality (mmol/L)				
Placebo	282 ± 4	289 ± 3^*^	286 ± 4^*^	0.034
Furosemide	282 ± 3	291 ± 3^*, †^	286 ± 3^*^	
p-total carbondioxide (mmol/L)				
Placebo	27 ± 2	25 ± 2^*^	25 ± 2^*^	< 0.001
Furosemide	26 ± 2	26 ± 2^†^	27 ± 2^†^	

*p-Na* Plasma concentrations of sodium, *p-K* potassium, *p-Cl* chloride and total carbondioxide and plasma osmolality were measured every 30 min during examination. Data show are values before hypertonic saline infusion, after 60 min of saline infusion, and 90 min after cessation of saline infusion on the examination day. Data are shown as medians with 25 and 75 percentiles in brackets. P-value represents probability of difference in response to saline (response from baseline to saline infusion) between treatments. To test difference in response to saline between treatments a students t-test was used. Wilcoxon signed rank test was performed to test differences from baseline, * = *p* < 0.05, and from Placebo, ^†^ = *p* < 0.05

P-K decreased in response to 3% saline and the decrease was more pronounced when furosemide was given. P-total carbon dioxide decreased in response to 3% saline but was unchanged in during furosemide. Responses in p-K and p-total carbon dioxide were significantly different after furosemide compared to placebo (Table 2). There was no correlation between the responses to 3% saline between p-Cl and p-total carbondioxide (*p* = 0.486) and p-K and p-total carbon dioxide (*p* = 0.895).

### GFR and tubular function during 3% saline and furosemide

Table 3 shows the effect of 3% saline and furosemide induced changes in GFR, urine output (UO), C_H2O_, u-Na (excretion rate), FE_Na_, FE_K_ and u-osmolality. Using a general linear model, expected different response patterns during both 3% saline and furosemide compared to 3% saline alone was demonstrated. UO decreased after 3% saline but increased markedly when saline infusion was accompanied by furosemide. In contrast GFR increased after 3% saline and decreased after furosemide treatment. C_H2O_ decreased after 3% saline but the decrease was initially less pronounced when furosemide was given.

**Table 3:** Effect of hypertonic saline and furosemide on GFR and tubular function in a randomized, cross-over study of 24 healthy subjects

Period	Baseline	Hypertonic saline infusion	Post hypertonic saline infusion				
	0–90 min	90–150 min	150–180 min	180–210 min	210–240 min	240–300 min	P (GLM within)
GFR (^51^Cr-EDTA clearance)							
Placebo	104 ± 14	102 ± 15	107 ± 15	110 ± 15^*^	111 ± 23^*^	112 ± 15^*^	0.001
Furosemide	104 ± 12	103 ± 13	103 ± 18	93 ± 14^*^	93 ± 14^*^	98 ± 12	
*P (GLM between)*	0.089						
Urine output (mL/min)							
Placebo	9.8 ± 1.5	3.5 ± 1.6^*^	2.7 ± 1.3^*^	2.7 ± 0.8^*^	3.3 ± 1.4^*^	4.7 ± 2.2^*^	< 0.001
Furosemide	9.1 ± 2.2	23.1 ± 2.6^*^	10.1 ± 3.1	4.3 ± 1.8^*^	2.4 ± 0.9^*^	2.0 ± 1.2^*^	
*P (GLM between)*	< 0.001						
C_H2O_ (ml/min)							
Placebo	6.6 ± 1.3	−0.4 ± 1.4^*^	−2.2 ± 1.2^*^	−2.4 ± 1.0^*^	−1.8 ± 1.6^*^	0.0 ± 2.1^*^	0.001
Furosemide	6.1 ± 2.0	1.1 ± 1.1^*^	−2.0 ± 0.7^*^	−1.8 ± 0.5^*^	−1.5 ± 0.5^*^	−1.1 ± 0.6^*^	
*P (GLM between)*	0.597						
U-Na (μmol/min)							
Placebo	200 ± 94	361 ± 146^*^	501 ± 234^*^	531 ± 182^*^	511 ± 161^*^	466 ± 106^*^	< 0.001
Furosemide	162 ± 78	2865 ± 342^*^	1515 ± 3.81^*^	659 ± 274^*^	377 ± 158^*^	273 ± 141^*^	
*P (GLM between)*	< 0.001						
FE_Na_ (%)							
Placebo	1.38 ± 0.63	2.46 ± 0.85^*^	3.28 ± 1.49^*^	3.36 ± 0.89^*^	3.26 ± 0.81^*^	3.00 ± 0.69^*^	< 0.001
Furosemide	1.13 ± 0.55	19.81 ± 3.11^*^	10.66 ± 3.81^*^	5.03 ± 1.97^*^	3.03 ± 1.59^*^	2.05 ± 1.31^*^	
*P (GLM between)*	< 0.001						
U-Cl (μmol/min)							
Placebo	239 ± 84	379 ± 146^*^	537 ± 261^*^	575 ± 201^*^	558 ± 185^*^	502 ± 122^*^	< 0.001
Furosemide	212 ± 61	3083 ± 356^*^	1679 ± 462^*^	763 ± 298^*^	441 ± 177^*^	310 ± 156^*^	
*P (GLM between)*	< 0.001						
FE_K_ (%)							
Placebo	21.1 ± 6.2	18.1 ± 7.1^*^	21.6 ± 17.0	23.1 ± 10.0	22.7 ± 8.0	21.9 ± 7.8	< 0.001
Furosemide	24.0 ± 9.2	64.9 ± 15.2^*^	44.6 ± 14.7^*^	34.6 ± 16.6^*^	27.4 ± 11.4	23.8 ± 10.5	
*P (GLM between)*	< 0.001						
U-osmolality (μmol//min)							
Placebo	899 ± 205	1103 ± 304^*^	1416 ± 592^*^	1485 ± 403^*^	1446 ± 351^*^	1351 ± 260^*^	< 0.001
Furosemide	831 ± 124	6293 ± 684^*^	3514 ± 881^*^	1746 ± 602^*^	1129 ± 323^*^	905 ± 328	
*P (GLM between)*	< 0.001						

*GFR* Glomerular filtration rate, urine output, *C*_*H2O*_ free water clearance, *u-Na/min* urinary sodium excretion, *FE*_*Na*_ fractional excretion of sodium, *u-Cl/min* urinary chloride excretion and *FE*_*K*_ fractional excretion of potassium, Urine was collected every 30 min in the 90 min baseline period, once after 60 min of hypertonic infusion, and every 30 min 90 min after hypertonic saline infusion and once 150 min after cessation of hypertonic saline infusion. Data from three baseline periods are pooled and shown as one period. Data are presented as means ± SD. Statistics are performed with a general linear model (GLM) or paired t-test. Difference from baseline: * = *p* < 0.05

U-Na, FE_Na_, u-Cl, and u-osmolality increased in response to saline and placebo and the increase was sustained throughout the examination. The increase was markedly pronounced when furosemide was given instead of placebo. After furosemide, the increases in u-Na, FE_Na_, u-Cl, and u-osmolality were however not sustained during the examination and decreased towards baseline values although it was still significantly higher in the last clearance period compared to baseline.

FE_K_ decreased slightly during 3% saline infusion. After infusion FE_K_ returned to baseline level. As expected furosemide increased FE_K_ with a substantial rapid response that declined during the clearance periods. The increase was maintained until the last two clearance periods.

### Markers of kidney injury

**Fig. 1:**
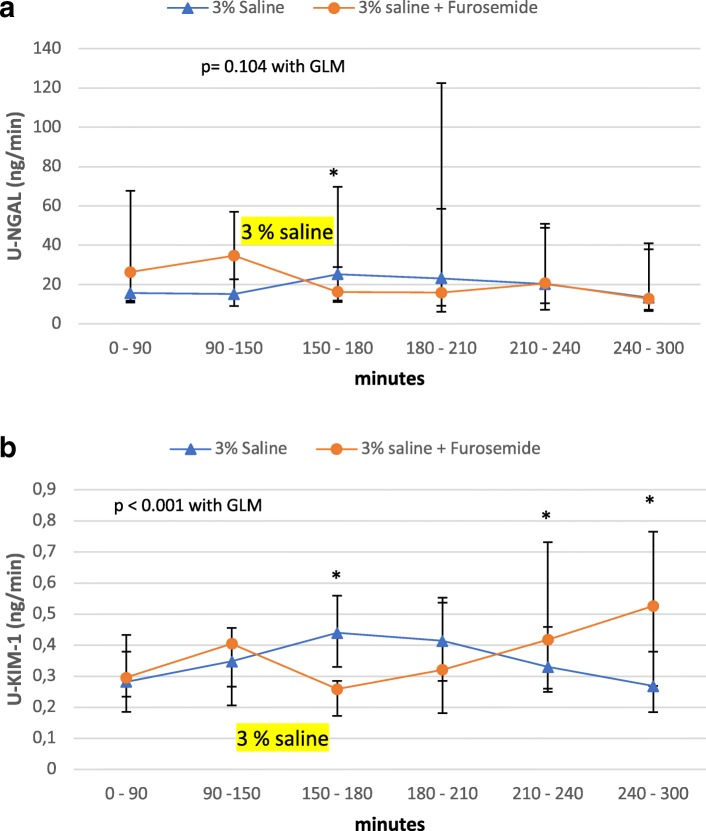
Effect of saline and furosemide on urinary excretion rate of neutrophil gelatinase-associated lipocalin (NGAL) (**a**) and kidney injury molecule − 1 (KIM-1) (**b**) in a randomized cross-over study of 24 healthy subjects. Urine was collected every 30 min in the 90 min baseline period, once after 60 min of 3% saline infusion, and every 30 min 90 min after hypertonic saline infusion and once 150 min after cessation of hypertonic saline infusion. Data from three baseline periods are pooled and shown as one period. Data are shown as medians with 25 and 75 percentiles in brackets. *P*-value represents probability of difference in response to hypertonic saline (response from baseline to hypertonic saline) between treatments Statistics are performed with a general linear model (GLM), and data were logarithmic transformed before GLM was performed. Difference in response from baseline between treatments are marked with * if *p* <  0.05 with a student’s t-test

**Fig. 2:**
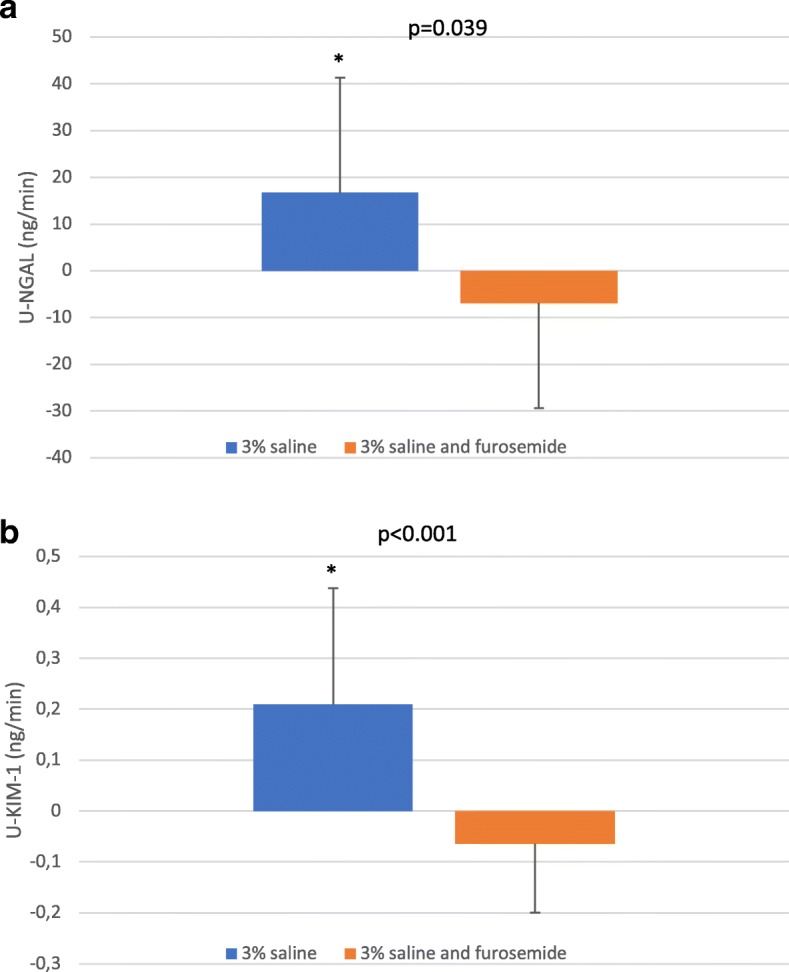
Change from baseline in urinary excretion rate of neutrophil gelatinase-associated lipocalin (NGAL) (**a**) and kidney injury molecule-1 (KIM-1) (**b**) in a randomized cross-over study of 24 healthy subjects. Values represent changes form baseline (0–90 min) to the period just after 3% saline infusion (150–180 min). The highest increase in u-NGAL and u-KIM-1 after 3% saline and placebo was observed in this period. Data are shown as means ± SD. P-value represents difference in response between treatments. * = *p* <  0.05 vs baseline. Statistics are performed a paired t-test

U-NGAL and u-KIM-1 excretion rates were similar between examination days at baseline (Fig. 1). U-NGAL increased slightly after 3% saline and placebo with a significant increase from baseline in the clearance period just after saline infusion was stopped (Fig. 2a, *p* = 0.034). In this period where the highest level of u-NGAL during placebo was observed, the response from baseline was significantly different from the response in furosemide group (Fig. 2a). However, when the entire examination was examined there was no difference in response between placebo and furosemide (*p* = 0.104 using GLM).

U-KIM-1 increased after 3% saline and placebo in the two clearance periods (150–210 min) following 3% saline infusion (Fig. 2b, *p* <  0.05). In the period from 150 to 180 min u-KIM-1 levels were highest and there was a significant difference in response from baseline compared with furosemide (Fig. 2b).

During furosemide no immediately increase in u-KIM-1 was observed, but u-KIM-1 increased in the last two clearance periods compared to placebo for both periods (Period 210–240 min: − 0.15 ± 0.18 in placebo vs. 0.21 ± 0.20 in furosemide, *p* <  0.001; Period 240–300 min: − 0.13 ± 0.12 vs. 0.14 ± 0.14, *p* <  0.001. Using a GLM the response in u-Kim-1 after 3% saline was significantly changed by furosemide (*p* <  0.001).

When u-NGAL and u-KIM-1 were adjusted for urinary creatinine excretion similar result as excretion rate were observed (data not shown).

### ENaC, AQP2 and UAER

Table 4 shows the effect of 3% saline and furosemide induced changes in u-AQP2, u-ENaC_γ_ and and UAER. U-AQP2 increased after 3% saline, and the increase was present after saline infusion was stopped. The response in u-AQP2 to 3% saline was changed by furosemide. U-AQP was markedly increased after furosemide during saline infusion compared to placebo. The following periods u-AQP2 decreased to baseline levels. U-ENaC_γ_ decreased to the same extent after 3% saline infusion in the two groups. UAER was not changed by 3% saline or the combination with furosemide.

**Table 4:** Effect of hypertonic saline and furosemide on excretion of proteins from epithelial sodium channels and aquaporin-2 channels in a randomized, cross-over study of 24 healthy subjects

Period	Baseline	Hypertonic saline infusion	Post hypertonic saline infusion				
	0–90 min	90–150 min	150–180 min	180–210 min	210–240 min	240–300 min	P (GLM within)
U-AQP2 (ng/minute)							
Placebo	0.81 (0.66;0.93)	0.85 (0.71;1.05)	1.00 (0.81;1.31)^*^	1.01 (0.87;1.38)^*^	1.07 (0.77;1.26)^*^	0.98 (0.80;1.09)^*^	< 0.001
Furosemide	0.77 (0.66;0.92)	1.42 (1.18;1.61)^*^	1.12 (0.90;1.41)^*^	1.14 (0.88;1.46)^*^	0.86 (0.72;1.10)^*^	0.79 (0.72;0.93)	
*P (GLM between)*	0.553						
U-AQP2 /creatinine (ng/mmol)							
Placebo	72 (66;84)	84 (74;87)	86 (83;104)^*^	102 (85;111)^*^	90 (77;99)^*^	92 (82;98)^*^	< 0.001
Furosemide	76 (63;83)	140 (104;150)^*^	100 (91;129)^*^	121 (84;132)^*^	97 (81;107)^*^	88 (64;104)^*^	
*P (GLM between)*	0.186						
U-ENaC_γ_ (ng/minute)							
Placebo	0.87 (0.71;1.27)	0.73 (0.60;1.19)	0.90 (0.76;1.27)	0.81 (0.70;1.10)	0.75 (0.64;1.13)	0.68 (0.60;1.03)^*^	0.399
Furosemide	0.92 (0.83;1.26)	0.87 (0.75;1.03)	1.06 (0.63;1.31)	0.83 (0.64;1.12)	0.81 (0.64;1.04)^*^	0.73 (0.63;0.93)^*^	
*P (GLM between)*	0.806						
U-ENaC_γ_ /creatinine (ng/mmol)							
Placebo	80 (72;97)	80 (63;90)	84 (72;98)	79 (63;87)	70 (63;89)	69 (62;81)^*^	0.884
Furosemide	91 (82;99)	75 (66;131)	81 (70;116)	88 (72;16)	83 (69;107)^*^	72 (60;97)^*^	
*P (GLM between)*	0.487						
UAER (μg/min)							
Placebo	1 (0;5)	3 (3;4)	4 (4;6)	4 (3;6)	4 (3;4)	3 (0;4)	0.129
Furosemide	1 (0;5)	0 (0;9)	0 (0;10)	4 (1;7)	4 (2;6)	3 (2;5)	
*P (GLM between)*	0.167						

*u-AQP2/minute* Aquaporin-2 excretion rate, *U-AQP2/creatinine* creatinine adjusted u-AQP2 excretion, *u-ENaC*_*γ*_*/minute* excretion of the γ-fraction of the epithelial sodium channel and *U-ENaC*_*γ*_ */creatinine* creatinine adjusted u-ENAC_γ_, *UAER* urinary albumin excretion rate. Urine was collected every 30 min in the 90 min baseline period, once after 60 min of hypertonic infusion, and every 30 min 90 min after hypertonic saline infusion and once 150 min after cessation of hypertonic saline infusion. Data from three baseline periods are pooled and shown as one period. Data are shown as medians with 25 and 75 percentiles in brackets. *P*-value represents probability of difference in response to hypertonic saline (response from baseline to hypertonic saline) between treatments Statistics are performed with a general linear model (GLM), or Wilcoxon signed rank test. Data were logarithmic transformed before GLM was performed. Difference from baseline: ^*^ = *p* < 0.05

### Vasoactive hormones in plasma

Plasma-AVP, PRC, p-Ang II and p-Aldo were similar at baseline (Table 5). 3% saline significantly increased AVP and the increased was more pronounced when furosemide was given with 3% saline. 3% saline significantly decreased PRC, p-AngII and p-Aldo. The responses in PRC, p-Ang II and p-Aldo to 3% saline were all significantly attenuated by furosemide.

**Table 5:** Effect of hypertonic saline and furosemide on vasoactive hormones in a randomized, cross-over study of 24 healthy subjects

	Baseline (90 min)	After 60 min hypertonic saline infusion (150 min)	90 min post hypertonic saline infusion (210 min)	P-value (difference in response)
p-AVP (ng/L)				
Placebo	0.20 (0.20;0.20)	0.50(0.40;0.70)^*^	0.20(0.20;0.23)	< 0.001
Furosemide	0.20 (0.18;0.20)	0.90(0.60;1.10)*^,†^	0.30(0.20;0.40)^*,†^	
PRC (ng/L)				
Placebo	9.0 (5.3;13.0)	7.3 (4.4;10.9)^*^	5.6 (2.9;7.4)^*^	0.001
Furosemide	10.3 (5.8;16.9)	9.3 (7.7;16.2)^†^	8.0 (5.3;19.3)^†^	
p-AngII (ng/L)				
Placebo	12 (8;18)	7 (5;13)^*^	6 (4;11) ^*^	0.014
Furosemide	16 (9;22)	16 (11;20)^†^	16 (9;24)^†^	
p-Aldo (pmol/L)				
Placebo	240 (200;342)	167 (144;211)^*^	169 (161;213)^*^	0.001
Furosemide	277 (232;377)	256 (228;328)^†^	262 (201;325)^†^	

*p-AVP* Plasma concentrations arginine vasopressin, *PRC* renin, *p-AngII* angiotensin II and *p-Aldo* aldosterone were measured before hypertonic saline infusion, after 60 min of saline infusion, and 90 min after cessation of saline infusion on the examination day. Data are shown as medians with 25 and 75 percentiles in brackets. *P*-value represents probability of difference in response to saline (response from baseline to saline infusion) between treatments. Students t-test was used to test difference in response to saline between treatments. Wilcoxon signed rank test was used to test statistical significant difference from baseline, ^*^ = *p* < 0.05, and from Placebo, ^†^ = *p* < 0.05

### Blood pressure (BP)

Hemodynamic variables are shown in Table 6. Systolic BP (SBP) was not altered by 3% saline, but diastolic BP (DBP) decreased. Furosemide changed the responses. When furosemide was given along with 3% saline SBP decreased. DBP also decreased but decreased but the response seemed delayed compared to placebo.

**Table 6:** Effect of hypertonic saline and furosemide on hemodynamic variables in a randomized, cross-over study of 24 healthy subjects

Period	Baseline	Hypertonic saline infusion	Post hypertonic saline infusion				
	0–90 min	90–150 min	150–180 min	180–210 min	210–240 min	240–300 min	P (GLM within)
SBP (mmHg)							
Placebo	118 ± 9	118 ± 10	117 ± 9	119 ± 10	117 ± 10	120 ± 9	0.001
Furosemide	117 ± 7	120 ± 13	114 ± 6^*^	111 ± 8^*^	113 ± 8^*^	116 ± 8	
*P (GLM between)*	0.202						
DBP (mmHg)							
Placebo	68 ± 7	67 ± 7	64 ± 8^*^	67 ± 7^*^	66 ± 6^*^	67 ± 8	0.003
Furosemide	68 ± 6	69 ± 7	67 ± 8	66 ± 6^*^	67 ± 7^*^	66 ± 6^*^	
*P (GLM between)*	0.740						
HR (beats/min)							
Placebo	62 ± 10	66 ± 11^*^	63 ± 11	64 ± 10^*^	62 ± 10	64 ± 11^*^	0.354
Furosemide	61 ± 8	65 ± 10^*^	63 ± 9^*^	63 ± 8^*^	64 ± 10^*^	64 ± 10^*^	
*P (GLM between)*	0.875						

*SBP, DBP* Systolic and diastolic blood pressure, *HR* heart rate, *cSBP, cDBP* central systolic and diastolic blood pressure, *AI* augmentation index *VR* vascular resistance. Blood pressure was measured every 30 min in the 90 min baseline period, once after 60 min of hypertonic infusion, and every 30 min 90 min after hypertonic saline infusion and once 150 min after cessation of hypertonic saline infusion. Data from four baseline measurements are pooled and shown as one period. Data are presented as means ± SD. Statistics are performed with a general linear model (GLM) or paired t-test. Statistically significant difference from baseline: * = *p* < 0.05

### Urinary spot samples day 1 and day 3–5 post examination

The results from urinary spot samples are shown in Table 7. The urinary spot sample performed 2 days after examination showed a decreased sodium concentration (u-Na) and increased potassium (u-K), creatinine and albumin concentration after furosemide compared to placebo (Table 7). Urine osmolality was increased after furosemide. Urinary chloride concentration (u-Cl), u-NGAL, u-KIM-1, u-AQP2 and u-ENaC_γ_ were not significally different.

**Table 7:** Effect of hypertonic saline and furosemide on urinary electrolytes and proteins in two spot urinary sample after examination in a randomized, cross-over study of 24 healthy subjects

	Spot 1 (day 1 post examination)	Spot 2 (day 3–5 post examination)
U-Na (mmol/L)		
Placebo	79 (41;105)	50 (31;135)
Furosemide	57 (27;98)^†^	62 (32;151)^*^
U-K (mmol/L)		
Placebo	26 (15;37)	27 (14;44)
Furosemide	34 (19;52)^†^	30 (17;58)^*^
U-Cl (mmol/L)		
Placebo	78 (53;126)	63 (40;129)
Furosemide	67 (38;116)	70 (45;186)
U-Creatinine (mmol/L)		
Placebo	4 (3;6)	5 (4;16)
Furosemide	8 (4;13)^†^	5 (3;13)^*^
U-Osmolality (mmol/L)		
Placebo	392 (200;467)	269 (195;710)^*^
Furosemide	430 (236;663)^†^	358 (214;740)^*^
U-Albumin (mg/L)		
Placebo	2 (1;5)	4 (2;7)
Furosemide	5 (3;6)^†^	4 (2;4)
U-ENaC_γ_ (ng/ml)		
Placebo	0.29 (0.18;0.44)	0.35 (0.19;0.85)
Furosemide	0.57 (0.27;0.92)	0.37 (0.21;0.92)
U-AQP2 (ng/ml)		
Placebo	0.40 (0.19;0.49)	0.37 (0.26;0.81)^*^
Furosemide	0.68 (0.27;0.95)	0.36 (0.29;1.08)^*^
U-NGAL (ng/ml)		
Placebo	8.5 (3.8;23.8)	9.5 (2.8;22.3)^*^
Furosemide	19.5 (4.0;40.5)	14.0 (3.0;25.5)^*^
U-KIM (ng/ml)		
Placebo	0.13 (0.08;0.17)	0.22 (0.09;0.35)^*^
Furosemide	0.40 (0.09;0.57)	0.20 (0.08;0.49)^*^

*u-Na* Urinary concentrations of sodium, *u-K* potassium, *u-Cl* chloride, creatinine, albumin, *u-ENaC*_*γ*_ γ-fraction of the epithelial sodium channel, *u-AQP2* aquaporin 2, *u-NGAL* neutrophil gelatinase-associated lipocalin and *u-KIM-1* kidney injury, molecule-1. Data are shown as medians with 25 and 75 percentiles in brackets. Wilcoxon signed rank test was used to test statistically significant difference from spot 1, * = *p* < 0.05, and from Placebo, ^†^ = *p* < 0.05

The urinary spot sample performed 3–5 days after examination, revealed no difference between the furosemide and placebo treatment for any of the variables in Table 7.

## Discussion

The main findings in this study was small increases in u-NGAL and U-KIM-1 after 3% saline. The increase in u-NGAL after 3% saline was abolished by furosemide. The response in u-KIM-1 was changed after furosemide, where the increase in u-KIM-1 after 3% saline was delayed to the last clearance periods. In addition, when furosemide was given along with 3% saline the increased p-Cl was attenuated and the decrease in p-total carbon dioxide was abolished. Although the increases in u-NGAL and u-KIM-1 after 3% saline were small, the increases may support the hypothesis that sodium-chloride solutions are nephrotoxic, but this study does not show convincing evidence for nephroprotective properties of furosemide.

Chloride induced metabolic acidosis after 0.9% saline (isotonic) has been reported previously [4, 6, 8–10, 31, 32]. The hyperchloremic acidosis is at least partly explained by intracellular displacement of the anion bicarbonate by chloride to reduce the anion gap in case of hyperchloremia [33]. A similar finding is also reported after hypertonic saline in healthy subjects where 3% saline increased plasma chloride and caused a respiratory compensated metabolic acidosis [34]. These findings were confirmed in our study were 3% saline infusion increased plasma chloride and evidence of acidosis was suggested by the reduced p-total carbondioxide. Total carbon dioxide is generally a good marker of serum bicarbonate due to the fact that bicarbonate comprises about 95% of total carbondioxide [6]. It is possible that the changes in total carbondioxide were due to changes in other forms of carbondioxide such as dissolved CO_2_ or carbonic acid, but most likely the changes are caused by changes in plasma bicarbonate. Furosemide attenuated the increase in plasma chloride and abolished the decrease in total carbondioxide after 3% saline. Assuming that total carbondioxide is a marker of bicarbonate, furosemide seems to prevent the metabolic acidosis induced by 3% saline. Metabolic alkalosis due to increased renal bicarbonate excretion is a known adverse reaction after furosemide treatment, although the renal mechanisms are not fully understood [35].

We measured two novel markers of kidney injury in the urine, NGAL and KIM-1, that are related to increased risk of renal replacement therapy and CKD in in patients with AKI [15–19]. Both u-NGAL and u-KIM-1 were slightly but significantly increased by 3% saline, suggesting renal injury induced by the hypertonic saline load. 3% saline increased GFR and decreased UO, which could influence the increase, but the increase was present when excretion was adjusted for urinary volume (flow) and creatinine excretion, so it is unlikely that that changes in GFR and UO are the explanation for the increased u-NGAL and u-KIM-1. Urine composition changed as expected after furosemide, with an increased osmolality and excretion of sodium and chloride, and these changes could have influenced the excretion of u-NGAL and u-KIM-1 without any kidney injury. However, it is unknown if marked changes in tubular electrolyte composition can change the excretion of u-NGAL and u-KIM-1. In spontaneously hypertensive rats high salt intake increased urinary NGAL and KIM-1 indicating that high dietary salt induces kidney injury [36]. High salt in this rat model was accompanied by an increased BP which is also likely to explain the increased urinary excretion of markers in kidney injury rather than salt intake itself. In the present study the salt load seemed to decrease BP rather than increase excluding blood pressure as a mediator of the increase in markers of kidney injury. Chloride and hyperchloremic acidosis has previously been demonstrated to influence renal hemodynamics by impairing RBF [9–11]. This is in contrast to observations in patients with heart failure where hypertonic saline preserved renal function, but no biomarkers were measured in these patients [37]. It is possible that certain patient groups may benefit from hypertonic saline while other patient groups does not. Patients with heart failure tend to be hypotensive and theoretically a volume expansion with 3% saline may increase blood pressure end subsequently RBF. We did not measure RBF and cannot evaluate changes in RBF. GFR was initially unchanged after 3% saline but increased in the last clearance periods which does not support a lowered RBF after 3% saline.

The loop-diuretic furosemide markedly increased UO and electrolyte excretion which was expected [20, 21, 23, 38, 39]. Furosemide attenuated the 3% saline induced increase in p-Cl and abolished the reduction in total carbondioxide. Hence furosemide attenuated the metabolic acidosis induced by 3% saline. The increases in u-NGAL and u-KIM-1, which were observed in the clearance periods just after 3% saline infusion, were abolished by furosemide. This might suggest renoprotective properties of furosemide. However, the increase in u-NGAL after 3% saline only just reached statistical significance and may be influenced by the huge increase in diuresis during furosemide, which dilutes the concentration of u-NGAL which increases the uncertainty of measurement. In addition, the increase in u-KIM-1 seems delayed after 3% saline and furosemide compared to placebo and was present in the last to clearance periods rather than the periods immediately after saline infusion. Accordingly, furosemide changed the response in u-KIM-1 where when compared to placebo a delayed increase was observed. It still under debate if furosemide is harmful or protective to the kidneys. Furosemide is shown to increase oxidative stress in the kidneys. [40] A recent meta-analysis did not find evidence of increased risk of AKI when furosemide was given as bolus injections [41]. In intensive care units furosemide is shown not to influence u-NGAL levels or renal prognosis [42, 43]. Although this study demonstrates some signs of positive protective effects of furosemide, further studies are warranted before conclusions can be drawn whether furosemide have harmful or protective properties after saline infusion.

AQP2 is located in the collecting duct principal cells and when inserted in the apical membrane increases water permeability and reabsorption [44]. AVP stimulates this insertion. Due to an increase in plasma osmolality induced by 3% saline the increases in AVP and subsequent increase in u-AQP2 were expected [20, 23]. The increase in AVP and u-AQP2 was further increased when furosemide was given simultaneously, likely explained by diuresis induced intravascular fluid depletion. Increased AVP and u-AQP2 to furosemide are established, and an additive increase in AVP due to the combined effects of 3% saline and furosemide was expected [21, 38, 39]. Hence 3% saline, furosemide and the combination of the two interventions induce increased water-reabsorption in the collecting ducts.

The 3% saline increased plasma osmolality and intravascular volume, and in concordance with our previous studies decreases in PRC, p-AngII and p-Aldosterone [20, 21, 23, 38, 39]. Furosemide caused a decrease in BP probably explained by a diuresis induced intravascular fluid depletion. Similarly, the decrease in the vasoactive hormones PRC, p-AngII and P-Aldo was attenuated and the increase in p-AVP was exaggerated. We have previously demonstrated that fluid depletion induced by furosemide creates increases in concentrations of PRC, p-AngII and p-Aldo [20, 21, 23, 38, 39]. This compensatory response is confirmed in this study where PRC, p-AngII and p-Aldo also increased after furosemide compared to placebo.

ENaC regulates sodium transport in the distal tubulus. In animal models changes in renal and plasma osmolality changed ENaC abundance in the collecting duct and ENaC activity [45, 46]. In previous studies small increases in u-ENaC_γ_ were observed in response to 3% saline [20, 23]. Hence we expected increases in u-ENaC_γ_ but in this study u-ENaC_γ_ was not changed by 3% saline. ENaC’s activity is regulated by aldosterone [47]. In this study p-Aldo decreased after 3% saline and was unchanged when furosemide was added, which can explain why u-ENaC_γ_ was unchanged. In addition, we used a higher infusion rate of 3% saline than used in previous studies resulting in a higher total dose of 3% saline, which could explain difference from previous studies of u-ENaC_γ_.

Despite being on an identically standardized diet 4 days prior to each examination there was a small but significantly lower sodium excretion in the 24-h prior to the examination where furosemide was given. All other parameters measured in the 24-h urine were not significantly different between examination days. This difference in sodium excretion may have influenced our results but we think it is unlikely because sodium excretion was similar at baseline on examinations days. The urinary spot samples collected day 1 after examination show furosemide changes in urine osmolality, creatinine, potassium and sodium concentration. These changes were not present in the spot urinary samples day 3–5 after examination. This suggest minimal carry-over effects of furosemide, which is a possibility in this cross-over study design.

There were no differences in markers of kidney injury in the post-experiment spot samples suggesting no long term nephrotoxic or nephroprotective effects of furosemide. The spot samples were collected at a random time between 7 AM and 2 PM and days without standardization of the diet, which could cause a larger variation in urine composition and we are therefore cautious to make definite conclusion based on these spot samples.

3% saline was chosen rather than 0.9% saline because we wanted to limit the confounding effects the volume load given with the saline infusion. Since 0.9% saline is mostly used in daily clinical settings and 3% saline is only used in specific cases, this reduces the generability to daily clinical practice. We chose the dose of 7 ml/kg/hour. This resulted in an average infusion dose of approximately 500 ml which we considered sufficient to give to see nephrotoxic effects of a high chloride load without safety concerns. The effect of different doses could reveal diffrences in urine excreation of renal injury but this needs further investigation.

## Conclusions

Furosemide given along with 3% saline attenuated the increase in p-Cl and prevented the decrease in p-total carbondioxide induced by 3% saline. The small increases in u-NGAL after 3% saline were abolished by furosemide. The increase in u-KIM-1 induced by hypertonic saline was delayed by furosemide Although the increases in u-NGAL and u-KIM-1 after 3% saline were small, the increases may support the hypothesis that sodium-chloride solutions are nephrotoxic. The changes i p-Cl, p-total carbon dioxide and u-NGAL suggest renoprotective properties as well, but the response in u-KIM-1 does not support this suggestion. Further investigations are warranted before conclusion can be made.

## Abbreviations

AKI: Acute kidney injury
Aldo: Aldosterone
AngII: Angiotensin II
AQP-2: aquaporin-2
AVP: vasopressin
BP: Blood pressure
C_H2O_: Free water clearance
CKD: Chronic kidney disease
Cl: Chloride
C_OSM_: Osmolar clearance
DBP: brachial diastolic blood pressure
EDTA: Ethylenediaminetetraacetic acid
ENaC_γ_: Gamma fraction of epithelial sodium channels
FE_K_: Fractional excretion of potassium
FE_Na_: Fractional excretion of sodium
GFR: Glomerular filtration rate
GLM: General linear model
HR: Heart rate
K: Potassium
KIM-1: kidney injury molecule-1
Na: Sodium
NGAL: Neutrophil gelatinase-associated lipocalin (u-NGAL)
PRC: Plasma concentration of renin
RBF: Renal blood flow
RIA: Radioimmunoassay
SBP: brachial systolic blood pressure
UAER: Urinary albumin excretion rate
UO: Urine output
